# Measurable residual disease at myeloablative allogeneic transplantation in adults with acute lymphoblastic leukemia: a retrospective registry study on 2780 patients from the acute leukemia working party of the EBMT

**DOI:** 10.1186/s13045-019-0790-x

**Published:** 2019-10-23

**Authors:** Jiří Pavlů, Myriam Labopin, Riitta Niittyvuopio, Gerard Socié, Ibrahim Yakoub-Agha, Depei Wu, Peter Remenyi, Jakob Passweg, Dietrich W. Beelen, Mahmoud Aljurf, Nicolaus Kröger, Hélène Labussière-Wallet, Zinaida Perić, Sebastian Giebel, Arnon Nagler, Mohamad Mohty

**Affiliations:** 1 Centre for Haematology, Imperial College London at Hammersmith Hospital, Du Cane Road, London, W12 0HS UK; 2 Department of Haematology, EBMT Paris Study Office/CEREST-TC/Saint Antoine Hospital, Paris, France; 3 Stem Cell Transplantation Unit, HUCH Comprehensive Cancer Center, Helsinki, Finland; 4 Hematology–BMT, Saint Louis Hospital, Paris, France; 5 Service des Maladies du Sang, Hopital Claude Huriez, CHRU Lille, Lille, France; 6 Department of Hematology, the First Affiliated Hospital of Soochow University, Soochow, China; 7 Délpesti Centrumkórház–Országos Hematológiai és Infektológiai Intézet, Budapest, Hungary; 8 University Hospital Basel, Basel, Switzerland; 9 Department of Bone Marrow Transplantation, University Hospital of Essen, Essen, Germany; 10 Department of Oncology, King Faisal Specialist Hospital and Research Center, Riyadh, Saudi Arabia; 11 Bone Marrow Transplantation Centre, University Hospital Eppendorf, Hamburg, Germany; 12 Service d’hématologie, Centre Hospitalier Lyon Sud, Lyon, France; 13 Zavod za hematologiju, Klinika za unutarnje bolesti, Zagreb, Croatia; 14 Maria Sklodowska-Curie Memorial Cancer Center and Institute of Oncology, Gliwice, Poland; 15 Division of Hematology and Bone Marrow Transplantation, Chaim Sheba Medical Center, Tel-Hashomer and Sackler School of Medicine, Ramat Gan, Israel

**Keywords:** Measurable residual disease, Allogeneic hematopoietic cell transplantation, Acute lymphoblastic leukemia, Allogeneic, Myeloablative conditioning, Total body irradiation

## Abstract

**Background:**

Assessment of measurable residual disease (MRD) is rapidly transforming the therapeutic and prognostic landscape of a wide range of hematological malignancies. Its prognostic value in acute lymphoblastic leukemia (ALL) has been established and MRD measured at the end of induction is increasingly used to guide further therapy. Although MRD detectable immediately before allogeneic hematopoietic cell transplantation (HCT) is known to be associated with poor outcomes, it is unclear if or to what extent this differs with different types of conditioning.

**Methods:**

In this retrospective registry study, we explored whether measurable residual disease (MRD) before allogeneic hematopoietic cell transplantation (HCT) for acute lymphoblastic leukemia is associated with different outcomes in recipients of myeloablative total body irradiation (TBI)-based versus chemotherapy-based conditioning. We analyzed outcomes of 2780 patients (median age 38 years, range 18–72) who underwent first HCT in complete remission between 2000 and 2017 using sibling or unrelated donors.

**Results:**

In 1816 of patients, no disease was detectable, and in 964 patients, MRD was positive. Conditioning was TBI-based in 2122 (76%) transplants. In the whole cohort MRD positivity was a significant independent factor for lower overall survival (OS) and leukemia-free survival (LFS), and for higher relapse incidence (RI), with respective hazard ratios (HR, 95% confidence intervals) of 1.19 (1.02–1.39), 1.26 (1.1–1.44), and 1.51 (1.26–1.8). TBI was associated with a higher OS, LFS, and lower RI with HR of 0.75 (0.62–0.90), 0.70 (0.60–0.82), and 0.60 (0.49–0.74), respectively. No significant interaction was found between MRD status and conditioning. When investigating the impact of MRD separately in the TBI and chemotherapy-based conditioning cohorts by multivariate analysis, we found MRD positivity to be associated with lower OS and LFS and higher RI in the TBI group, and with higher RI in the chemotherapy group. TBI-based conditioning was associated with improved outcomes in both MRD-negative and MRD-positive patients.

**Conclusions:**

In this large study, we confirmed that patients who are MRD-negative prior to HCT achieve superior outcomes. This is particularly apparent if TBI conditioning is used. All patients with ALL irrespective of MRD status benefit from TBI-based conditioning in the myeloablative setting.

## Background

Assessment of measurable residual disease (MRD) is rapidly transforming the therapeutic and prognostic landscape of a wide range of hematological malignancies. Its prognostic value in acute lymphoblastic leukemia (ALL) has been established and MRD measured post-induction or consolidation is increasingly used to guide further therapy [1].

The prognostic value of MRD measured prior to allogeneic hematopoietic cell transplantation (HCT) on its outcomes was first observed in small retrospective [2, 3] and prospective [4] studies of children and adolescents and later also in adults [5–7], and confirmed in a recent meta-analysis [8]. However, it remains unclear if or to what extent the choice of conditioning regimen impacts on this. We have recently studied the interaction of myeloablative versus reduced-intensity conditioning and MRD in acute myeloid leukemia [9]. As ALL patients rarely receive reduced-intensity conditioning, we explored if MRD detectable before allogeneic HCT for ALL is associated with different outcomes in recipients of myeloablative total body irradiation (TBI)-based versus chemotherapy-based conditioning.

## Methods

### Study design and data collection

This was a multicenter, retrospective registry analysis, approved by the Acute Leukemia Working Party of the European Society for Blood and Marrow Transplantation (EBMT). The EBMT is a voluntary group that represents more than 600 transplant centers, predominantly European. EBMT centers pay annual subscriptions to maintain the EBMT Registry.

EBMT Med A/B standardized data collection forms [10] are submitted to the registry by transplant center personnel following written informed consent from patients in accordance with center ethical research guidelines. Accuracy of data is assured by the individual transplant centers and by quality control measures such as regular internal and external audits. Presence of Philadelphia chromosome status was collected. The results of disease assessments at HCT were also submitted and form the basis of this report.

Eligibility criteria were age 18 years or older, a diagnosis of de novo ALL, disease status at transplant of morphological first complete remission supplemented by a report of MRD status, recipients of first myeloablative HCT during the study period 2000 to 2017, a stem cell source that was either unmanipulated peripheral blood stem cells or bone marrow and a donor that was a sibling or unrelated 9/10 or 10/10 matched. Table 1 provides numbers of patients fulfilling the inclusion criteria and availability of required information in the EBMT database. MRD methodology and allocation to MRD-negative or MRD-positive groups were determined by individual participating centers and utilized molecular and/or immunophenotyping criteria. An additional audit of methods used in the EBMT centers contributing to the study showed that 34 of 56 centers (61%) used both PCR-based and immunophenotyping-based techniques. PCR-based techniques only were used in 11 centers and immunophenotyping only also in 11 centers (19.6%). All centers but one regarded an MRD level of 10^−4^ or lower as negative (for one center this was less than 10^−3^). Intensity of conditioning was allocated in accordance with published criteria [11].

**Table 1:** Numbers of patients fulfilling the inclusion criteria with required information

Inclusion criteria	*N*
Adults with ALL in CR1 or CR2 allografted from MSD or UD 10/10 or UD 9/10 from January 2000 to December 2017	10,418
Myeloablative conditioning	8400
Available information	
Immunophenotype B or T and Philadelphia status	5540
MRD status before transplantation reported	2780

### Statistical methods

Measured outcomes were leukemia-free survival (LFS), relapse incidence (RI), non-relapse mortality (NRM), overall survival (OS), acute graft-vs-host disease (aGVHD), chronic graft-vs-host-disease (cGVHD), and GVHD-free and relapse-free survival (GRFS). LFS was defined as survival with no evidence of relapse or progression. Relapse was defined as a reappearance of blasts in the blood or bone marrow (> 5%) or in any extramedullary site. NRM was defined as death without evidence of relapse or progression. OS was defined as the time from HCT to death, regardless of the cause. GRFS was defined as survival free of events including grade 3–4 aGVHD, extensive cGVHD, relapse, or death [12].

Probabilities of OS, LFS, and GRFS were calculated using the Kaplan-Meier method. Cumulative incidence was used to estimate the endpoints of NRM, RI, aGVHD, and cGVHD to accommodate competing risks. To study aGVHD and cGVHD, we considered relapse and death to be competing risks. Univariate analyses were done using Gray’s test for cumulative incidence functions and the log-rank test for OS, GRFS, and LFS. A Cox proportional hazards model was used for multivariate regression. All variables differing significantly between the two groups or factors known to influence outcomes were included in the Cox model. In order to test for a center effect, we introduced a random effect or frailty for each center into the model [13, 14]. Results were expressed as the hazard ratio (HR) with the 95% confidence interval (95% CI). The type I error rate was fixed at 0.05 for the determination of factors associated with time-to-event outcomes.

After analysis of the whole group, two separate planned sub-analyses of TBI-based conditioning and chemotherapy only conditioning were made. Statistical analyses were performed with SPSS 24.0 (SPSS Inc., Chicago, IL) and R 3.4.1 (R Core Team 2017) [15].

## Results

### Demographics and transplant details

A total of 2780 patients from 301 transplant centers were eligible. Median age at transplantation was 38 years (range 18–72). In 1816 (65%) of patients, no disease was detectable, and in 964 (35%) patients, MRD was positive. Conditioning was TBI-based in 2122 (76%) transplants and chemotherapy-based in 658 (24%) transplants. Details of patient and transplant characteristics by MRD status are summarized in Table 2. More patients with Philadelphia chromosome-positive B-ALL were MRD-positive at transplantation (66 versus 49%, *P* < .001). Patients who were MRD-negative at the time of transplantation were less likely to receive donor lymphocytes after the procedure (7% versus 12%, *P* < .001). With a medium follow-up of 42 months the probability of OS, LFS, GRSF, and RI at 2 years for the whole cohort was 65% (95% CI 63–70), 55% (95% CI 53–57), 42% (95% CI 39–44), and 27% (95% CI 25–29), respectively.

**Table 2:** Demographics and transplant details

Characteristic	MRD negative	MRD positive	*P*
*N*	1816	964	
Median follow-up, (months, IQR)	39.70 (12.89–84.20)	44.56 (16.07–82.07)	0.410
Median age (years, range, IQR)	36 (18–70, 26–46)	38 (18–72, 28–48)	< 0.001
Median time dg to HCT (months, range, IQR)	5.7 (1.9–130, 4.6–8)	5.6 (2.3–123, 4.5–7.7)	0.182
Median year of HCT (range)	2012 (2000–2017)	2012 (2000–2017)	0.687
Median donor age (years, range, IQR, missing)	34 (4–73, 26–44, 643)	35 (10–72, 27–44, 293)	0.080
In vivo TCD			0.221
No in vivo TCD	1099 (62%)	565 (60%)	
In vivo TCD	670 (38%)	381 (40%)	
Data missing	47	18	
Remission status at HCT			0.518
CR1	1580 (87%)	847 (88%)	
CR2	236 (13%)	117 (12%)	
ALL subtype			< 0.001
B-ALL Ph-negative	479 (26%)	181 (19%)	
B-ALL Ph-positive	882 (49%)	639 (66%)	
T-ALL	455 (25%)	144 (15%)	
Karnofsky score at HCT			0.203
< 80%	60 (4%)	41 (5%)	
> =80%	1639 (96%)	861 (95%)	
Data missing	117	62	
Engraftment			0.459
Engrafted	1732 (98%)	925 (99%)	
Graft failure	29 (1.7%)	12 (1.3%)	
Data missing	55	27	
Source of stem cells			0.008
Bone marrow	409 (23%)	261 (27%)	
Blood	1407 (78%)	703 (73%)	
Donor type			0.268
Matched sibling	1041 (57%)	531 (55%)	
Unrelated 10/10 match	575 (32%)	308 (32%)	
Unrelated 9/10 match	200 (11%)	125 (13%)	
Conditioning			0.628
Chemotherapy-based	435 (24%)	223 (23%)	
TBI containing	1381 (76%)	741 (77%)	
Patient sex			0.285
Male	1097 (60%)	603 (63%)	
Female	717 (40%)	361 (37%)	
Data missing	2	0	
Donor sex			0.267
Male	1107 (62%)	606 (64%)	
Female	693 (39%)	346 (36%)	
Data missing	16	12	
Donor–recipient sex mismatch			0.411
Female to male	391 (22%)	195 (20%)	
Other	1409 (78%)	762 (80%)	
Data missing	16	7	
Patient CMV serology			0.950
Negative	637 (37%)	342 (37%)	
Positive	1090 (63%)	582 (63%)	
Data missing	89	40	
Donor CMV serology			0.690
Negative	790 (46%)	414 (45%)	
Positive	927 (54%)	502 (55%)	
Data missing	99	48	
CMV donor/recipient			0.975
Negative to negative	450 (27%)	239 (27%)	
Positive to negative	176 (10%)	99 (11%)	
Negative to positive	317 (19%)	166 (18%)	
Positive to positive	743 (44%)	398 (44%)	
Data missing	130	62	
HCT-comorbidity index			0.128
1 or 2	529 (85%)	271 (81%)	
> =3	92 (15%)	62 (19%)	
Data missing	1195	631	
GVHD prevention			0.054
Cyclosporin	124 (7%)	60 (6%)	
Cyclosporin and MTX	1247 (70%)	702 (74%)	
Cyclosporin and MMF ± MTX	181 (10%)	101 (11%)	
Tacrolimus ± other	115 (7%)	41 (4%)	
Other	103 (6%)	41 (4%)	
Data missing	46	19	
Acute GVHD			0.139
Grade 0–I	1156 (67%)	594 (64%)	
Grade II–IV	564 (33%)	329 (36%)	
Data missing	96	41	
Donor lymphocyte infusion			< 0.001
None received	1681 (93%)	846 (88%)	
Pre-emptive	36 (2%)	48 (5%)	
After relapse	97 (5%)	67 (7%)	
Data missing	2	3	

*Abbreviations*: *CR* complete remission, *CMV* cytomegalovirus, *GVHD* graft-versus-host disease, *HCT* hematopoietic cell transplantation, *IQR* interquartile range, *dg* diagnosis, *MMF* mycophenolate mofetil, *MTX* methotrexate; *MRD* measurable residual disease, *Ph* Philadelphia chromosome/BCR-ABL gene rearrangement, *TCD* T cell depletion

### Univariate analysis

Compared to MDR-negative status MRD-positive status at the time of transplantation was associated with significantly worse probability of OS (61% versus 67%), LFS (50% versus 58%), GRFS (35% versus 45%), and with higher RI (32% versus 24%) at 2 years post-transplantation. The full results of univariate analysis are summarized in Additional file 2.

### Multivariate analysis

The results of multivariate analysis by Cox regression showed MRD positivity was a significant independent factor for lower survival and LFS, and for higher RI, with respective HR of 1.19 (95% CI 1.02–1.39), 1.26 (95% CI 1.1–1.44), and 1.51 (95% CI 1.26–1.8). Of the potentially modifiable factors, use of TBI-based conditioning was associated with a higher OS, LFS, and lower RI with HR of 0.75 (95% CI 0.62–0.90), 0.70 (95% CI 0.60–0.82), and 0.60 (95% CI 0.49–0.74), respectively. Use of in vivo T cell depletion was associated with decreased NRM, improved GRFS, lower incidence acute grade II–IV, grade III–IV, chronic, and extensive chronic GVHD, with HR of 0.68 (95% CI 0.52–0.88), 0.75 (95% CI 0.64–0.88), 0.72 (95% CI 0.59–0.89), 0.51 (95% CI 0.35–0.75), 0.58 (95% CI 0.47–0.71), and 0.48 (95% CI 0.36–0.64), respectively. The prognostic impact of MRD status did not differ significantly according to the conditioning. Results of multivariate analysis of the whole cohort are summarized in Table 3.

**Table 3:** Multivariate analysis of factors determining outcomes at 2 years

*N* = 2156	RI		NRM		LFS		OS		GRFS		Acute GVHD II-IV		Acute GVHD III-IV		Chronic GVHD		Extensive cGVHD	
	HR (95% CI)	*P*	HR (95% CI)	*P*	HR (95% CI)	*P*	HR (95% CI)	*P*	HR (95% CI)	*P*	HR (95% CI)	*P*	HR (95% CI)	*P*	HR (95% CI)	*P*	HR (95% CI)	*P*
MRD pos vs neg	1.51 (1.26–1.80)	< 0.001	0.99 (0.80–1.23)	0.928	1.26 (1.10–1.44)	0.001	1.19 (1.02–1.39)	0.028	1.25 (1.10–1.41)	< 0.001	1.12 (0.96–1.32)	0.161	1.09 (0.80–1.48)	0.585	1.00 (0.85–1.18)	0.996	0.99 (0.79–1.24)	0.949
Ph neg B-ALL	1	–	1	–	1	–	1	–	1	–	1	–	1	–	1	–	1	–
Ph pos B-ALL	0.94 (0.75–1.18)	0.609	1.43 (1.07–1.91)	0.015	1.12 (0.94–1.33)	0.198	0.94 (0.72–1.14)	0.531	1.02 (0.87–1.19)	0.818	0.96 (0.79–1.17)	0.663	0.87 (0.61–1.25)	0.456	0.95 (0.78–1.16)	0.637	1.08 (0.82–1.42)	0.578
T-ALL	1.11 (0.86–1.43)	0.445	1.11 (0.78–1.58)	0.554	1.13 (0.92–1.39)	0.246	1.06 (0.85–1.33)	0.611	1.03 (0.86–1.25)	0.720	1.10 (0.87–1.39)	0.429	0.84 (0.54–1.30)	0.440	0.84 (0.66–1.06)	0.138	1.01 (0.73–1.41)	0.938
Age (per 10 years)	1.03 (0.96–1.11)	0.369	1.32 (1.22–1.42)	< 0.001	1.15 (1.09–1.21)	< 0.001	1.21 (1.14–1.28)	< 0.001	1.12 (1.06–1.18)	< 0.001	1.08 (1.01–1.15)	0.019	1.09 (0.97–1.22)	0.170	1.07 (1.01–1.14)	0.025	1.06 (0.97–1.15)	0.218
Year of HCT	0.97 (0.95–0.99)	0.042	0.97 (0.94–1.00)	0.053	0.97 (0.95–0.99)	0.005	0.98 (0.95–0.10)	0.030	0.99 (0.97–1.00)	0.096	0.98 (0.96–0.10)	0.032	0.97 (0.93–1.01)	0.173	0.97 (0.94–0.99)	0.002	1.01 (0.98–1.04)	0.628
CR2 vs CR1	2.29 (1.83–2.88)	< 0.001	1.63 (1.2–2.23)	0.002	2.02 (1.68–2.42)	< 0.001	2.11 (1.72–2.59)	< 0.001	1.70 (1.43–2.03)	< 0.001	1.21 (0.97–1.52)	0.096	1.58 (1.06–2.36)	0.025	1.07 (0.82–1.40)	0.604	1.19 (0.82–1.73)	0.358
KPS > =90%	1.09 (0.88–1.35)	0.418	1.14 (0.89–1.47)	0.297	1.12 (0.95–1.31)	0.173	1.01 (0.85–1.21)	0.896	1.03 (0.90–1.19)	0.648	0.98 (0.82–1.18)	0.839	0.97 (0.69–1.36)	0.841	1.02 (0.85–1.22)	0.826	1.08 (0.84–1.39)	0.543
UD 10/10	0.66 (0.52–0.83)	< 0.001	1.91 (1.45–2.51)	< 0.001	1.02 (0.86–1.22)	0.793	1.24 (1.01–1.51)	0.038	1.14 (0.97–1.34)	0.125	1.66 (1.35–2.05)	< 0.001	2.02 (1.38–2.95)	< 0.001	1.39 (1.14–1.70)	0.001	1.26 (0.96–1.66)	0.099
UD 9/10	0.57 (0.42–0.81)	0.001	2.10 (1.49–2.98)	< 0.001	1.01 (0.80–1.27)	0.942	1.31 (1.01–1.69)	0.043	1.02 (0.82–1.26)	0.876	1.73 (1.33–2.26)	< 0.001	1.75 (1.04–2.94)	0.035	1.32 (1.01–1.73)	0.042	1.07 (0.73–1.59)	0.716
Blood vs BM	0.90 (0.73–1.10)	0.288	1.07 (0.83–1.38)	0.607	0.95 (0.82–1.11)	0.534	0.39 (0.77–1.12)	0.432	1.17 (1.01–1.36)	0.043	1.08 (0.88–1.33)	0.450	1.14 (0.79–1.65)	0.496	1.44 (1.18–1.76)	< 0.001	1.83 (1.37–2.45)	< 0.001
Female vs male	0.80 (0.67–0.96)	0.018	0.93 (0.75–1.16)	0.531	0.86 (0.75–0.99)	0.031	0.86 (0.74–1.01)	0.062	0.88 (0.77–0.99)	0.039	0.94 (0.80–1.11)	0.466	0.73 (0.53–0.10)	0.047	0.92 (0.79–1.08)	0.294	0.91 (0.73–1.14)	0.411
Donor fem vs male	0.66 (0.55–0.80)	< 0.001	1.29 (1.05–1.60)	0.018	0.89 (0.77–1.02)	0.098	0.97 (0.83–1.13)	0.671	0.96 (0.85–1.09)	0.519	1.11 (0.94–1.30)	0.209	1.00 (0.73–1.37)	0.986	1.33 (1.13–1.55)	< 0.001	1.23 (0.99–1.53)	0.0593
Pt CMV pos vs neg	0.93 (0.76–1.13)	0.447	1.23 (0.98–1.56)	0.081	1.06 (0.91–1.23)	0.462	1.24 (1.05–1.47)	0.014	1.02 (0.89–1.17)	0.784	0.96 (0.80–1.13)	0.603	1.01 (0.73–1.41)	0.943	0.10 (0.84–1.19)	0.972	1.04 (0.81–1.32)	0.780
Dr CMV pos vs neg	1.12 (0.92–1.36)	0.276	0.89 (0.71–1.11)	0.300	0.99 (0.86–1.15)	0.931	0.95 (0.81–1.12)	0.548	1.07 (0.94–1.23)	0.315	1.08 (0.91–1.28)	0.364	1.11 (0.80–1.54)	0.523	1.18 (1.00–1.40)	0.0567	1.30 (1.02–1.65)	0.036
TBI vs chemo	0.60 (0.49–0.74)	< 0.001	0.87 (0.68–1.12)	0.292	0.70 (0.60–0.82)	< 0.001	0.75 (0.62–0.90)	0.002	0.88 (0.76–1.03)	0.104	1.18 (0.95–1.46)	0.126	1.02 (0.69–1.51)	0.922	1.23 (0.99–1.52)	0.0647	1.27 (0.93–1.75)	0.131
In vivo TCD vs no TCD	1.22 (0.97–1.53)	0.090	0.68 (0.52–0.88)	0.004	0.94 (0.79–1.12)	0.494	0.84 (0.69–1.02)	0.077	0.75 (0.64–0.88)	< 0.001	0.72 (0.59–0.89)	0.002	0.51 (0.33–0.75)	< 0.001	0.58 (0.47–0.71)	< 0.001	0.48 (0.36–0.64)	< 0.001
Center (frailty)	–	0.314	–	0.17	–	0.295	–	0.015	–	0.019	–	< 0.001	–	< 0.001	–	0.028	–	0.004

*Abbreviations*: *BM* bone marrow, *CR* complete remission, *CMV* cytomegalovirus, *dr* donor, *GVHD* graft-versus-host disease, *GRFS* GVHD-free and relapse-free survival, *HCT* hematopoietic cell transplantation, *KPS* Karnofsky performance score, *LFS* leukemia-free survival, *MRD* measurable residual disease, *NRM* non-relapse mortality, *OS* overall survival, *Ph* Philadelphia chromosome/BCR-ABL gene rearrangement, *pt* patient, *RI* relapse incidence, *TCD* T cell depletion, *UD* unrelated donor

**Fig. 1:**
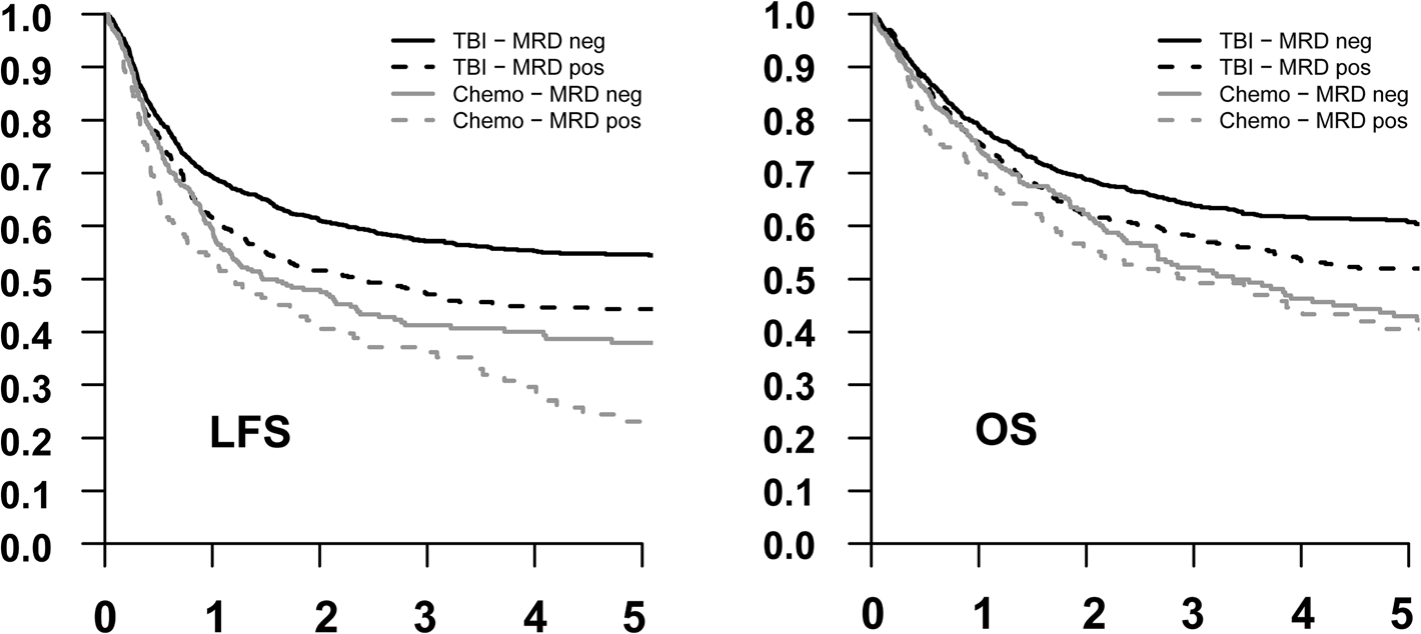
Survival of 2780 adults transplanted for ALL after myeloablative conditioning. Kaplan-Meier curves show estimates of leukemia-free survival (LFS, left) and overall survival (OS, right). Curves for patients with undetectable MRD (MRD neg) at transplantation are shown in full lines, and for MRD-positive (MRD pos) patients in broken lines. Curves related to TBI-based conditioning are shown black and to chemotherapy-based conditioning in gray lines

When investigating the impact of MRD separately in the TBI and chemotherapy-based conditioning cohorts by multivariate analysis, we found MRD positivity to be associated with lower OS and LFS and higher RI in the TBI group, and with higher RI in the chemotherapy group (results are summarized in Additional file 3). TBI-based conditioning was associated with improved outcomes in both MRD-negative and MRD-positive patients (Fig. 1).

## Discussion

In this large study, we confirmed that adult patients with ALL who are MRD-negative prior to allogeneic HCT achieve superior outcomes, namely, lower RI, higher LFS, and OS. We were interested in exploring potential differing outcomes between recipients of TBI-based conditioning and conditioning based on chemotherapy only. While TBI-based conditioning is associated with significant short as well as long-term toxicity [16], it remains part of most conditioning protocols for ALL because it is believed to have a better anti-leukemic potential in lymphoid malignancies. In animal experiments, administration of high doses of busulfan had little impact on lymphoid organs [17] or on antibody responses [18]. In children, a small randomized trial [19] showed better event-free survival with TBI-based regiments, and a recent large international randomized trial closed, after an interim analysis showed a survival benefit in patients who received TBI-based conditioning over chemotherapy-based conditioning [20]. There are no such randomized prospective trials in adults, but data in many retrospective studies, including recently published large analysis by the EBMT suggested advantages of TBI-based over chemotherapy-based regimens, particularly in terms of reduced risk of relapse and improved LFS [21]. This effect was also seen in adults transplanted for primary refractory ALL [22] with a large tumor bulk as well as in patients with T-ALL, regardless of their remission status [23]. So far, however, the impact of conditioning has not been studied in the context of MRD. It has been unclear if TBI is necessary for patients who achieved MRD negativity as a graft-versus-leukemia effect may be sufficient to eliminate very low level of residual disease.

This study showed significantly superior outcomes with the use of TBI-based conditioning in both MRD-positive and MDR-negative patients, but the impact of MRD did not differ significantly between the TBI-based or chemotherapy-based conditioning. MRD positivity was associated with lower OS and LFS and higher RI in the larger (*n* = 1943) TBI subgroup, and with higher RI in the smaller (*n* = 571) chemotherapy subgroup. The reasons for this cannot be concluded from this study, but it is possible that ALL cells are able to escape the effect of chemotherapy in sanctuary sites such as CNS, and/or that the ALL is simply more susceptible to effects of radiotherapy. No patients received radiotherapy before starting transplantation conditioning, so irradiation represents a different anti-leukemic treatment modality to chemotherapy in patients transplanted after TBI-based conditioning. Also, patients in this cohort did not receive modern immunotherapy such as inotuzumab, ozogamicin, or blinatumomab that are able to induce MRD negativity on their own [24, 25] or in addition to chemotherapy [26, 27]. It is likely that with the use of these agents, more patients may become MRD-negative. Whether they will or will not benefit from TBI-based conditioning as the MRD-negative patients in this cohort remains unclear, but clinicians should not rush into rejecting TBI-based conditioning in patients with ALL.

Compared to related donors, unrelated donors both 10/10 and 9/10 had a lower incidence of relapse. This suggests better anti-leukemic activity and increased GVHD with lower degree of histocompatibility. Unlike in recent studies of T cell-replete haploidentical transplantation with post-transplantation cyclophosphamide [28, 29], this increase in anti-leukemic activity did not improve OS due to higher incidence of aGVHD, cGVHD, and NRM.

Interestingly, in vivo T cell depletion was associated with higher RI, lower NRM, and lower incidence of aGVHD and cGVHD only in patients who received TBI-based, but not chemotherapy-based conditioning. This phenomenon may suggest more profound immune allogeneic effect in conjunction with the use of TBI-based conditioning, perhaps due to more significant lymphodepletion seen in animal experiments after TBI but not after chemotherapy [17, 18]. Some previous publications suggested an increased incidence of GVHD after TBI-based conditioning [30, 31], but there is also data in contrary to this [32]. Surprisingly, in the chemotherapy-based, but not TBI-based conditioning subgroup, MRD-positive patients experienced higher RI, but comparable LFS and OS. Although it is possible to speculate that patients who relapsed after chemotherapy-based conditioning benefited more from salvage treatments with donor lymphocytes, the difference may be also due to the size of the groups and resulting statistical power.

Although the majority of EBMT centers use highly sensitive methods of MDR detection [33], and our an additional audit showed that all 56 centers but 1 regarded an MRD level of 10^−4^ or lower as negative (for one center this was less than 10^−3^), an obvious limitation of this registry study is the lack of access to details of MRD methodologies and targets used in individual patients. However, the proportion of reported MRD-positive cases seen was 35% of the total eligible for the study and this is similar to the 21 to 38*%* reported in studies where detailed review of MRD methodology and targets were feasible [34, 35]. Centers were required to declare the MRD status of patients prior to HCT, but we did not have access to the precise timing of the relevant MRD assay. Another important issue is potential heterogeneity of conditioning regimens within the TBI and chemotherapy groups [36].

The challenge of how best to manage MRD positivity pre-HCT in the clinic is a familiar dilemma since further therapy may incur toxicity that renders subsequent HCT undeliverable or may result in frank relapse should the leukemia show resistance to the new treatment modality. In the post-HCT setting, management of MRD-positive patients has involved strategies such as rapid withdrawal of immunosuppressive medication, pre-emptive use of donor lymphocyte infusions, and maintenance therapy with tyrosine kinase inhibitors in Philadelphia-positive patients. In the future, immunotherapy such as blinatumomab [37], chimeric antigen receptor T cells, natural killer cells, or check-point inhibitors may be useful mostly in patients with B cell ALL.

## Conclusions

In this large study, we confirmed that adult patients with acute lymphoblastic leukemia who are MRD-negative prior to HCT achieve superior outcomes. This was particularly apparent with the use of TBI-based conditioning. With increasing availability of new therapies MRD negativity is likely to become achievable for more patients, hopefully leading to improved treatment outcomes. As all patients with ALL irrespective of MRD status benefit from TBI-based conditioning, avoidance of it on the basis of achievement of MRD negativity is not justified.

## Supplementary information

**Additional file 1.** List of all institutions reporting data included in this study.

**Additional file 2.** Univariate analysis of factors determining outcomes of transplantation at 2 years.

**Additional file 3.** A. Univariate planned sub-analyses performed separately in subgroups of patients transplanted after chemotherapy-based and TBI-based conditioning. Abbreviations: GVHD, graft-versus-host disease; GRFS, GVHD-free and relapse-free survival; LFS, leukemia free survival; MRD, measurable residual disease; NRM, non-relapse mortality; OS, overall survival; RI, relapse incidence. B. Multivariate planned sub-analyses performed separately in subgroups of patients transplanted after chemotherapy-based conditioning (571 patients of whom 382 were MRD negative and 205 MRD positive) and TBI-based conditioning (1943 patients of whom 1278 were MRD negative and 680 MRD positive). Abbreviations: BM, bone marrow; CR, complete remission; CMV, cytomegalovirus; GVHD, graft-versus-host disease; GRFS, GVHD-free and relapse-free survival; KPS, Karnofsky performance score; LFS, leukemia free survival; MRD, measurable residual disease; NRM, non-relapse mortality; OS, overall survival; Ph, Philadelphia chromosome/BCR-ABL gene rearrangement; RI, relapse incidence; TCD, T-cell depletion; UD, unrelated donor.

## Data Availability

The dataset supporting the conclusions of this article are available in the ALWP of EBMT in Paris, 184 rue Faubourg Saint Antoine.

## Abbreviations

aGVHD: Acute graft-versus-host disease
ALL: Acute lymphoblastic leukemia
ALWP: Acute leukemia working party
cGVHD: Chronic graft-versus-host-disease
CI: Confidence interval
EBMT: European Society for Blood and Marrow Transplantation
GRFS: GVHD-free and relapse-free survival
GVHD: Graft-versus-host-disease
HCT: Hematopoietic cell transplantation
HR: Hazard ratio
LFS: Leukemia-free survival
MRD: Measurable residual disease
NRM: Non-relapse mortality
OS: Overall survival
RI: Relapse incidence
TBI: Total body irradiation
