# Supplementary material for: Measurable residual disease at myeloablative allogeneic transplantation in adults with acute lymphoblastic leukemia: a retrospective registry study on 2780 patients from the acute leukemia working party of the EBMT
Source: J Hematol Oncol. 2019 Oct 23;12:108. doi: 10.1186/s13045-019-0790-x (PMC6813121; doi:10.1186/s13045-019-0790-x)
Supplement: Supplementary file 1 — Additional file 1. List of all institutions reporting data included in this study. [file 13045_2019_790_MOESM1_ESM.docx]

Additional file 1**:** List of all institutions reporting data included in this study.

Mazzoni Hospital, Haematology Service, Ascoli Piceno, Italy; USD Trapianti di Midollo, Adulti, Pizzale Spedali Civili 1, Brescia, Italy; Universitaetsmedizin Mannheim, III. Medizinische Klinik, Mannheim, Germany; Robert Bosch Krankenhaus, Abt. Hämatologie/Onkologie, Stuttgart, Germany; Klinikum Augsburg, II Medizinische Klinik, Augsburg, Germany; Asklepios Klinik St.Georg, Department of Haematology, Hamburg, Germany; Hôpital Necker, Service Hematologie Adulte, Paris, France; Tel Aviv Sourasky Medical Center, Blood and Bone Marrow Transplantation, Tel Aviv, Israel; Hospital Guglielmo da Saliceto, Oncology and Hematology Department, Piacenza, Italy; Hacettepe University, Department of Hematology, Ankara, Turkey; Gazi University Faculty of Medicine, Hematology, Ankara, Turkey; Centre National de Greffe de Moelle, Rue Jebel Lakhdar, Tunis, Tunisia; Adnan Menderes University Med. Faculty, Hematology Department, Aydin, Turkey; Klinikum Frankfurt (Oder) GmbH, Medizinische Klinik I, Frankfurt Oder, Germany; Haukeland University Hospital, Department of Haematology, Bergen, Norway; University Hospital, Hematology, Basel, Switzerland; Leiden University Hospital, BMT Centre Leiden, Leiden, Netherlands, The; Klinik fuer Innere Medzin III, Universitätsklinikum Ulm, Ulm, Germany; Imperial College, Department of Haematology, London, United Kingdom; Bone Marrow Transplant Unit L 4043, National University Hospital, Copenhagen, Denmark; Hopital St. Louis, Dept.of Hematology - BMT, Paris, France; University Hospital, Clinic of Hematology, Zurich, Switzerland; University Hospital Gasthuisberg, Dept. of Hematology, Leuven, Belgium; Hospital Sirio-Libanes, Hematology Bone Marrow Transplant Unit, Sao Paulo, Brazil; Karolinska University Hospital, Dept. of Hematology, Stockholm, Sweden; Hospital Clinic, Institute of Hematology & Oncology, Barcelona, Spain; Institut Jules Bordet, Experimental Hematology, Brussels, Belgium; Ospedale San Martino, Department of Haematology II, Genova, Italy; Royal Marsden Hospital, Leukaemia Myeloma Units, London, United Kingdom; Universitaet Tuebingen, Medizinische Klinik, Tuebingen, Germany; University College London Hospital, Department of Haematology, London, United Kingdom; Turku University Hospital, TD7 (Stem Cell Transplant Unit), Turku, Finland; Medizinische Universitaet Wien, Klinik fuer Innere Medizin I, Vienna, Austria; Western General Hospital, Dept. of Haematology, Edinburgh, United Kingdom; Programme de Transplantation & Therapie Cellulaire, Centre de Recherche en Cancérologie de Marseille, Marseille, France; S.S.C.V.D Trapianto di Cellule Staminali, A.O.U Citta della Salute e della Scienza di Torino, Torino, Italy; Univ. La Sapienza, Dip. Biotecnologie Cellulari ed Ematologia, Rome, Italy; Hopital Jean Minjoz, Service d`Hématologie, Besancon, France; Cliniques Universitaires St. Luc, Dept. of Haematology, Brussels, Belgium; Oslo University Hospital, Rikshospitalet, Clinic for Cancer Medicine, Hematology Dept., Oslo, Norway; Hospital de la Princesa, Department of Hematology, Madrid, Spain; Hosp. Reina Sofia, Córdoba Hospital, Cordoba, Spain; University Medical Centre, Dept. of Haematology, Utrecht, Netherlands, The; Bologna University, S.Orsola-Malpighi Hospital, Institute of Hematology & Medical, Bologna, Italy; Hospital U. Marqués de Valdecilla, Servicio de Hematología-Hemoterapia, Santander, Spain; Bone Marrow Transplant Unit, Beatson, West of Scotland Cancer Centre, Glasgow, United Kingdom; Univ. of Parma, Cattedra di Ematologia, Parma, Italy; Erasmus MC Cancer Institute, University Medical Center Rotterdam, Rotterdam, Netherlands, The; Ospedale Civile, Dipartimento di Ematologia, Medicina Trasfusionale e Biotecnologie, Pescara, Italy; Institut de Cancerologie Lucien Neuwirth, 108 B avenue Albert Raimond, Saint Etienne, France; CHU CAEN, Institut d’hématologie de Basse-Normandie, Caen, France; Hôpital Henri Mondor, Sve d` Hematologie, Creteil, France; CHU Nantes, Dept. D`Hematologie, Nantes, France; Yorkshire Blood & Marrow Transplant Programme, Haematology Department, Level 3, Leeds, United Kingdom; Department of Haematology, Cancer and Haematology Centre, Oxford, United Kingdom; University Medical Center Schleswig-Holstein, Campus Kiel, División of Stem Cell Transplantation and Immunotherapy, Kiel, Germany; Hope Directorate, St. James`s Hospital, Dublin, Ireland; Hadassah University Hospital, Dept. of Bone Marrow Transplantation, Jerusalem, Israel; University Hospital, Dept. of Bone Marrow Transplantation, Essen, Germany; Hospital Santa Creu i Sant Pau, Hematology Department, Barcelona, Spain; Département d`Oncologie, Service d`Hématologie, Hôpitaux Universitaires De Genève, Geneva, Switzerland; Universite Paris IV, Hopital la Pitié-Salpêtrière, Hematologie Clinique, Paris, France; The Trustee of London Clinic, Stem Cell Transplant Unit, London, United Kingdom; Hopital La Miletrie, Head of the Bone Marrow TransplantUnit, Poitiers, France; Fondazione IRCCS - Ca’ Granda, Ospedale Maggiore Policlinico IRCCS, Milano, Italy; University Hospital, Dept. of Medicine, Uppsala, Sweden; CHU Bordeaux, Hôpital Haut-leveque, Pessac, France; Belfast City Hospital, Dept. of Haematology, Belfast, United Kingdom; CHU Grenoble Alpes - Université Grenoble Alpes, Service d`Hématologie, Grenoble, France; University Hospital Innsbruck, Internal Medicine V (Hematology & Oncology), Innsbruck, Austria; Hopital Bretonneau, Service d`Oncologie Médicale, Tours, France; CHU ESTAING, Service d’hématologie clinique Adulte et pédiatrie, Clermont Ferr, France; Adult HSCT unit, Northern Centre for Bone Marrow Transplantation, Newcastle Tyne, United Kingdom; CHU de Lille, LIRIC, INSERM U995, Lille, France; Centro Trapianti di Midollo Osseo, Clinica Pediatrica Università di Milano Bicocca, Monza, Italy; University Hospital of Patras, Internal Medicine / BMT Unit, Patras, Greece; Hospital Clínico Universitario, Servicio de Hematología, Valencia, Spain; Skanes University Hospital, Dept. of Hematology, Lund, Sweden; Birmingham Heartlands Hospital, Department of Haematology, Birmingham, United Kingdom; BMT unit, Clinica Ematologica, Pavia, Italy; Ospedale S. Camillo-Forlanini, Dept. of Hematology and BMT, Rome, Italy; Sahlgrenska University Hospital, Center for Hematopoietic Cell Transplantation, Goeteborg, Sweden; Klinikum Karlsruhe gGmbH, III. Med. Klinik, Karlsruhe, Germany; Inst. Português de Oncologia do Porto, BMT Unit, Porto, Portugal; ASST GRANDE OSPEDALE METROPOLITANO NIGUARDA, Hematology Department, Milano, Italy; Hannover Medical School, Department of Haematology, Hemostasis, Oncology, Hannover, Germany; Goethe-Universitaet, Medizinische Klinik II, Hämatologie, Medizinische Onkologie, Frankfurt Main, Germany; Hospital San Maurizio, Dept. of Hematology - BMT Unit, Bolzano, Italy; Inst. Portugues Oncologia, BMT Unit, Lisboa, Portugal; Hopital d`Enfants de la Timone, CHU, Département Hématologie Oncologie Pédiatrique, Marseille, France; University Hospital Center Rebro, Kispaticeva 12, Zagreb, Croatia; Department of Haematology, University Hospital of Wales, Cardiff, United Kingdom; Azienda Ospedaliera Universitaria Careggi, Cell Therapy and Transfusion Medicine Unit, Firenze, Italy; Onco-Ematologia Pediatrica, Centro Trapianti Cellule Staminali, Torino, Italy; Universita Cattolica S. Cuore, Istituto di Ematologia, Rome, Italy; Medical University Graz, LKH - University Hospital Graz, Graz, Austria; Fundación Jiménez Díaz, Hematología, Madrid, Spain; Deutsche Klinik fuer Diagnostik, KMT Zentrum, Wiesbaden, Germany; U.O.S.A Centro Trapianti e Terapia Cellulare, Azienda Ospedaliera Universitaria Senese, Siena, Italy; Hospital Universitario Virgen de la Arrixaca, Ctra. Madrid - Cartagena, Murcia, Spain; European Institute of Oncology, Institute of Haematology, Milano, Italy; Ospedale Nord, Institute of Haematology, Taranto, Italy; ZNA, Lange Beeldekensstraat 267, Antwerp, Belgium; Emergency Care Centre, Aberdeen Royal Infirmary, Aberdeen, United Kingdom; Rambam Medical Center, Dept. of Hematology & BMT, Haifa, Israel; University Hospital &quot;Queen Johanna-Isul&quot;, 8, `Bialo More` Str., Sofia, Bulgaria; University Hospital Aachen, Dept. of Oncology, Hematology and SCT, Aachen, Germany; GHDC, Department of Hemato-Oncology, Charleroi, Belgium; Istituto Clinico Humanitas, Transplantation Unit, Milano, Italy; Gemeinnützige Salzburger, Landeskliniken Betriebsges.m.b.H., Salzburg, Austria; Universitaetsklinium Magdeburg, Med. Fakultät d., Magdeburg, Germany; Complejo Hospitalario de A Coruña, Hematologia (Planta 11ª), La Coruna, Spain; Department of Internal Medicine, American University of Beirut Medical Center, Beirut, Lebanon; Univ. Est. de Campinas/TMO/UNICAMP, Cidade Universitaria `Zeferino Vaz`, Campinas, Brazil; Bristol Royal Hospital for Children, Dept. of Paediatric Oncology/BMT, Bristol, United Kingdom; University Hospital Birmingham NHSTrust, Queen Elizabeth Medical Centre, Edgbaston, Birmingham, United Kingdom; Medical Clinic and Policinic 1, Hematology and Cellular Therapy, Leipzig, Germany; Heinrich Heine Universitaet, Klinik für Hämat,Onkol,Klin.Immun., Duesseldorf, Germany; U.O.D Trapianti di midollo osseo, A.O.R Villa Sofia-Cervello, Palermo, Italy; King Faisal Specialist Hospital & Research Centre, Oncology (Section of Adult Haematolgy/BMT), Riyadh, Saudi Arabia; Bone Marrow Transplantation Center, The First Affiliated Hospital, Hangzhou, China; Gaziantep University Medical School, Division of Haematology, Gaziantep, Turkey; Beilinson Hospital, Hematology and BMT Department, Petach Tikva, Israel; Ankara Bayindir Hospital, Haematology BMT, Ankara, Turkey; Hospital Álvaro Cunqueiro - Complejo Hospitalario Universitario de Vigo, Servicio de Hematología, Vigo, Spain; Fundeni Clinical Institute, 258 Fundeni Street, Building A, 7th Floor, Bucharest, Romania; Department of Bone Marrow Transplantation and Oncohematology, Maria Sklodowska-Curie Institute, Gliwice, Poland; Anadolu Medical Center Hospital, Bone Marrow Transplantation Department, Kocaeli, Turkey; King Fahad Specialist Hospital, Adult Hematology and HSCT department, Dammam, Saudi Arabia; Peking University People´s Hospital, Institute of Haematology, No 11 Xizhimen South Street,, Beijing, China; King Abdul - Aziz Medical City, , Riyadh, Saudi Arabia; Istanbul Medipol University,, Medipol Mega Hospital Complex, Istanbul, Turkey; Istanbul Medipol University,, Medipol Mega Hospital Complex, Istanbul, Turkey; University Hospital Motol, Department of Paediatric Haematology And Oncology, Prague, Czech Republic; Albert Alberts Stem Cell Transplantation Centre, Netcare Pretoria East Hospital, Pretoria, South Africa; Clatterbridge Cancer Centre - Liverpool, Royal Liverpool University Hospital, Clatterbridge Cancer Centre NHS Foundation Trust, Liverpool, United Kingdom; Ospedale Dell`Angelo, Hematology Department, Venezia, Italy; A.Z. Sint-Jan, Dept. of Hematology, Brugge, Belgium; Klinikum Grosshadern, Med. Klinik III, Munich, Germany; HUCH Comprehensive Cancer Center, Stem Cell Transplantation Unit, Helsinki, Finland; HELIOS Klinikum Berlin-Buch, Klinik für Hämatologie und Stammzelltransplantation, Berlin, Germany; CHU Nice - Hôpital de l`ARCHET I, Hematologie Clinique, Nice, France; University of Heidelberg, Medizinische Klinik u. Poliklinik V, Heidelberg, Germany; IRCCS, Casa Sollievo della Sofferenza, DEPARTMENT OF HEMATO-ONCOLOGY, S. Giovanni Rot, Italy; Pesaro Hospital, Hematology & Transplant Centre, Pesaro, Italy; Klinik fuer Innere Medizin C, Hämatologie und Onkologie, Transplantationszentrum, Greifswald, Germany; Universitaetsklinikum Jena, Klinik für Innere Medizin II, Jena, Germany; University of Cologne, I. Dept. of Medicine, Cologne, Germany; Hospital de Gran Canaria `Dr Negrin`, Servicio de Hematología y Hemoterapia, Las Palmas, Spain; DCTK, ul. Grabiszynska 105, Wroclaw, Poland; St. George`s Hospital, Department of Haematology, London, United Kingdom; Azienda Ospedaliero Universitaria di Modena Policlinico, Ematologia, Modena, Italy; Ospedale San Gerardo, Clinica Ematologica dell`Universita Milano-Biocca, Monza, Italy; University Medical Center Groningen (UMCG), Dept. of Hematology, Groningen, Netherlands, The; Universitaetsklinikum Goettingen, Abteilung Hämatologie und Onkologie, Goettingen, Germany; Jagiellonian University, Department of Haematology, Krakow, Poland; Dél-pesti Centrumkórház –, Országos Hematológiai és Infektológiai Intézet, Budapest, Hungary; Fondazione IRCCS Policlinico San Matteo, Pediatric Hematology-Oncology, Pavia, Italy; Klinikum Rechts der Isar, III Med Klinik der TU, Munich, Germany; Hospital Univ. Virgen de las Nieves, Servicio de Hematología, Granada, Spain; George Papanicolaou General Hospital, Haematology Department / BMT Unit, Thessaloniki, Greece; University Hospital Maastricht, Dept. Internal Med.Hematology /Oncology, Maastricht, Netherlands, The; Addenbrookes Hospital, Department of Haematology, Cambridge, United Kingdom; Hospital Clinico Universitario, Servicio de Hematología, S de Compostela, Spain; NADACE HAIMOM, University Hospital, Olomouc, Czech Republic; Unidad de Ensayos Clínicos de Hematología Pabellón A, bajo., Complejo Hospitalario de Navarra, Pamplona, Spain; King Hussein Cancer Centre, Queen Rania Street - Aljubiha, Amman, Jordan; Clinic of Hematology, Military Medical Academy, Belgrade, Serbia and Montenegro; National Haematology Centre, Clinic Linezers, Riga, Latvia; Hospital Vall d`Hebron, Unidad de Adultos, Barcelona, Spain; Universitaet Rostock, Kl. für Inn.Med./Hämatologie/Onkol., Rostock, Germany; Grande Ospedale Metropolitano Bianchi Melacrino Morelli - Centro Unico Trapianti A. Neri, Via G. Melacrino, 21, Reggio Calabria, Italy; VU University Medical Center, Department of Hematology (Br 250), Amsterdam, Netherlands, The; Charité - Campus Benjamin Franklin, Universitaetsmedizin Berlin, Berlin, Germany; Belarussian Research Center for Pediatric Oncology, Hematology and Immunology, Frunzenskaya str., 43, Minsk, Belarus; Klinik fuer Knochenmarktransplantation, und Hämatologie/Onkologie GmbH, Idar Oberstein, Germany; LKH - University Hospital Graz, Division of Paediatric Haemato/Oncology, Graz, Austria; Elisabethinen-Hospital, I. Internal Department, Linz, Austria; Malignant Haematology & Stem Cell Tranplantation, Alfred Hospital, Melbourne, Australia; University Hospital Brno, Dept. of Internal Med. - Hematooncology, Brno, Czech Republic; Hospital Universitario Donostia, Paseo Dr Beguiristain 107-116, San Sebastian, Spain; Manchester Royal Infirmary, Clinica Haematology Department, Manchester, United Kingdom; Klinikum Bremen-Mitte, Hämatologie / Onkologie, Bremen, Germany; Attikon University General Hospital, BMT Unit, 2nd Dept. of Internal Medicine, Athens, Greece; Az. Ospedaliera S. Croce e Carle, Division of Hematology, Cuneo, Italy; Hospital Ampang, Jalan Mewah Utara, Ampang, Malaysia; University Hospital, Department of Hematology and Transfusiology, Bratislava, Slovakia; Hospital del SAS, Dept. of Hematology, Cadiz, Spain; ICO-Hospital Universitari Germans Trias i Pujol, Cattedra e Servizio di Ematologia, Barcelona, Spain; University Hospital Eppendorf, Bone Marrow Transplantation Centre, Hamburg, Germany; Hospital Ramón y Cajal, Servicio de Hematología, Madrid, Spain; Ankara University Faculty of Medicine, Dept. of Hematology, Ankara, Turkey; Evangelismos Hospital, Division of Hematology, BMT Unit, Athens, Greece; Policlinico G.B. Rossi, Divisione di Ematologia, Unità di TMO, Verona, Italy; CHU - Institut Universitaire du Cancer Toulouse, Oncopole, Toulouse, France; Klinikum Nuernberg, 5. Medizinische Klinik, BMT-Unit, Nuernberg, Germany; Erciyes Medical School, Dept. of Hematology - Oncology, Kayseri, Turkey; Ege University Medical School, Dept. of Hematology, Izmir, Turkey; Shariati Hospital, Hematology-Oncology and BMT Research, Teheran, Iran; University Med. Center, Department of Hematology, Ljubljana, Slovenia; Hospital Universitario Central de Asturias, Avenida de Roma S/N, Oviedo, Spain; Vilnius University Hospital Santaros Klinikos, Haematology, Oncology & Transfusion Center, Vilnius, Lithuania; Philipps Universitaet Marburg, University Hospital Giessen and Marburg, Marburg, Germany; AZ Delta, Hematology - Oncology Dept., Roeselare, Belgium; U.O. Ematologia con Trapianto, Azienda Ospedaliero Universitaria Policlinico Bari, Bari, Italy; CHRU, Service des Maladies du Sang, Angers, France; Institute of Hematology and Blood Transfusion, Servicio de Hematología, Prague, Czech Republic; ASST Papa Giovanni XXIII, Hematology and Bone Marrow Transplant Unit, Bergamo, Italy; C.H.R.U de Brest, 2, avenue Foch, Brest, France; Arcispedale S. Maria Nuova, Unita Operativa Ematologia, Reggio Emilia, Italy; Centre Hospitalier Universitaire de Rennes, Service d`Hematologie Clinique Adulte, Rennes, France; Hôpital Percy, Hematology Department, Clamart, France; Gustave Roussy Cancer Campus, BMT Service, Department of Hematology,, Villejuif, France; Centre Hospitalier Lyon Sud, Pavillon Marcel Bérard -Bat 1G, Lyon, France; Techniciens d`Etude Clinique suivi de patients greffes, Nouvel Hopital Civil, Strasbourg, France; Hopital d`Enfants, Hematology, Vandoeuvre Nanc, France; Silesian Medical Academy, Univ. Dept. of Haematology and BMT, Katowice, Poland; University of Muenster, Dept. of Hematol./Oncol., Muenster, Germany; Pediatric University Teaching Hospital, BMT Unit, II Children`s Clinic, Bratislava, Slovakia; Osmangazi University, Fac. of Medicine, Hasan Dolatkan Street, Eskisehir, Turkey; Dokuz Eylul University, School of Medicine, Izmir, Turkey; Ankara Numune Education and, Research Hospital, Ankara, Turkey; Ospedale La Maddalena - Dpt. Oncologico, Unità Operativa di Oncoematologia e, Palermo, Italy; Institute of Hematology and Transfusion Medicine, I. Gandhi 14 str., Warsaw, Poland; Samodzielny Publiczny, Szpital Kliniczny Nr 1 w Lublinie, Lublin, Poland; University Hospital SPSK 1, Department of Hematology, Wroclaw, Poland; Centre Pierre et Marie Curie, Service Hématologie Greffe de Moëlle, Alger, Algeria; Southampton General Hospital, Haematology, Oncology, & Paediatrics, Southampton, United Kingdom; Azienda Ospedaliero Universitaria di Udine, Division of Hematology, Udine, Italy; Fiona Stanley Hospital, Hematology Department, Perth, Australia; Universitaetsklinikum Wuerzburg, Med. Klinik und Poliklinik II, Wuerzburg, Germany; Leicester Royal Infirmary, Department of Haematology, Leicester, United Kingdom; Nottingham University, Hucknall Road, Nottingham, United Kingdom; Charles University Hospital, Dept. of Hematology/Oncology, Pilsen, Czech Republic; Hospital Universitari Son Espases, Hematology Service, Palma Mallorca, Spain; First State Pavlov Medical University of St. Petersburg, Raisa Gorbacheva Memorial Research Institute for Paediatric Oncology, Hematology, and Transplantation, St Petersburg, Russia; University of Liege, Dept. of Hematology, Liege, Belgium; Hospital Clínico, Servicio de Hematología, Salamanca, Spain; Clinica Puerta de Hierro, Servicio de Hematologia y Hemoterapia, Madrid, Spain; Charles University Hospital, 4th Department of Internal Medicine - Hematology, Hradec Kralove, Czech Republic; Poznan University of Medical Sciences, Department of Hematology and Bone Marrow Transplantation, Poznan, Poland; Umea University Hospital, Hematology, Umea, Sweden; Hospital Morales Meseguer, C/ Marqués de los Velez s/n, Murcia, Spain; University Hospital, Dept. of Hematology, Linkoeping, Sweden; Ghent University Hospital, Haematology, Gent, Belgium; Tartu University Hospital, Clinic of Hematology and Oncology, Tartu, Estonia; Klinikum Oldenburg, Abt. Onkologie/Hämatologie, Oldenburg, Germany; Haematology Department, St.Savvas Oncology Hospital, Athens, Greece; Chaim Sheba Medical Center, Chaim Sheba Medical Center, Tel Hashomer, Israel; ¨Tor Vergata¨ University of Rome, Stem Cell Transplant Unit, Rome, Italy; ICO – Hospital Duran i Reynals, Av. Gran Vía 199-203, Barcelona, Spain; Ýstanbul Tip Fakultesi, Iç Hastaliklari ABD, Istanbul, Turkey; Istanbul University-Cerrahpasa, Cerrahpasa Medical Faculty BMT Unit, Istanbul, Turkey; GKT School of Medicine, Dept. of Haematological Medicine, London, United Kingdom; University of Napoli, `Federico II` Medical School, Napoli, Italy; Hospital Universitario Virgen del Rocío, Servicio de Hematologia y Hemoterapia, Sevilla, Spain; Hopital Saint Antoine, Department of Hematology, Paris, France; Sheffield Teaching Hospitals NHS Trust, South Yorkshire Region (Adult) BMT Programme, Sheffield, United Kingdom; Christie NHS Trust Hospital, Adult Leukaemia and Bone Marrow Transplant Unit, Manchester, United Kingdom; University of Saarland, University Hospital, Homburg, Germany; University Medical Center Mainz, , Mainz, Germany; University Regensburg, Dept. of Hematology and Oncology, Regensburg, Germany; Azienda Ospedali Riuniti di Ancona, Department of Hematology, Ancona, Italy; A.O.R.N. `SAN.G MOSCATI`, Ematologia, Avellino, Italy; Ospedale Policlinico, Programma di Trapianto Emopoietico Misto e Metropolitano Di Catania, Catania, Italy; Sezione di Ematologia, Dipartimento di Medicina Clinica e Sperimentale, Perugia, Italy; Azienda Ospedaliero Universitaria Pisana, Unità Operativa Ematologia, Pisa, Italy; IRRCS Ospedale Pediatrico Bambino Gesù, Piazza S. Onofrio, 4, Rome, Italy; S. Bortolo Hospital, Department of Hematology, Vicenza, Italy; Canterbury Health Laboratories, Department of Haematology, Christchurch, New Zealand; Medical University of Gdansk, Dept. of Haematology, Gdansk, Poland; Institut d`Hematologie et d`Oncologie Pediatrique, 1 Place Professeur Joseph Renaut, Lyon, France; Charité Universitaetsmedizin Berlin, Campus Virchow Klinikum, Berlin, Germany; Universitaetsklinikum Dresden, Medizinische Klinik und Poliklinik I, Dresden, Germany; University Hospital Erlangen, Dept. of Internal Medicine 5, Erlangen, Germany; University of Freiburg, Dept. of Medicine -Hematology, Oncology, Freiburg, Germany; Centro Trapianti Unico Di CSE Adulti e Pediatrico A. O Brotzu, Via Edward Jenner, 1, Cagliari, Italy; Ospedale San Raffaele s.r.l., Haematology and BMT, Milano, Italy; Military Institute of Health Services BMT Unit, Bone Marrow Transplantation Unit, Warsaw, Poland; Cape of Hope, Wroclaw Medical University, Wroclaw, Poland; Hospital Gregorio Marañón, Sección de Trasplante de Medula Osea, Madrid, Spain; University Hospitals Plymouth NHS Trust, Derriford Hospital, Plymouth, United Kingdom; H SS. Antonio e Biagio, Haematology Department, Alessandria, Italy; King Faisal Specialist Hospital and Research Center, Department of Oncology, Jeddah, Saudi Arabia; National Hospital of Haematological Diseases, Bone Marrow Transplant, Sofia, Bulgaria; Ospedale San Carlo, Dip. Ematologia, Potenza, Italy; Private Medicana International Ankara Hospital, Dept. Bone Marrow Transplantation, Ankara, Turkey; Medical Park Hospitals, Stem Cell Transplant Unit, Antalya, Turkey; Federal Centre of Heart, Blood and Endocrinology, named after V.A. Almazov, St Petersburg, Russia; CHU Lapeyronie, Département d`Hématologie Clinique, Montpellier, France; Medical School University of Salerno, AOU San Giovanni di Dio e Ruggi D´Aragona Hospital, Salerno, Italy; National Research Center for Hematology, Bone Marrow Transplantation, Moscow, Russia; First Affiliated Hospital of Soochow University, Department of Hematology, Suzhou, China; Centre Henri Becquerel, Hematology, Rouen, France; Central Clinical Hospital, The Medical University of Warsaw, Warsaw, Poland; University of Amiens: CHU Amiens, Service d`Hematologie, Amiens, France; St. Franziskus Hospital, Medizinische Klinik I, Flensburg, Germany; CHRU Limoges, Service d`Hématologie Clinique, Limoges, France; FOSCAL-UNAB, Urbanización El Bosque, Floridablanca, Colombia; Florence Nightingale Sisli Hospital, Hematopoietic SCT Unit, Istanbul, Turkey; Antwerp University Hospital (UZA), Dept. of Hematology, Antwerp Edegem, Belgium.
