# Supplementary material for: Measurable residual disease at myeloablative allogeneic transplantation in adults with acute lymphoblastic leukemia: a retrospective registry study on 2780 patients from the acute leukemia working party of the EBMT
Source: J Hematol Oncol. 2019 Oct 23;12:108. doi: 10.1186/s13045-019-0790-x (PMC6813121; doi:10.1186/s13045-019-0790-x)
Supplement: Supplementary file 2 — Additional file 2. Univariate analysis of factors determining outcomes of transplantation at 2 years. [file 13045_2019_790_MOESM2_ESM.docx]

Additional file 2:

Univariate analysis of factors determining outcomes of transplantation at 2 years. Abbreviations: BM, bone marrow; CR, complete remission; CMV, cytomegalovirus; GVHD, graft-versus-host disease; GRFS, GVHD-free and relapse-free survival; KPS, Karnofsky performance score; LFS, leukemia free survival; MRD, measurable residual disease; MSD, matched sibling donor; NRM, non-relapse mortality; OS, overall survival; Ph, Philadelphia chromosome/BCR-ABL gene rearrangement; RI, relapse incidence; TCD, T-cell depletion; UD, unrelated donor.

| **Univariate analysis** |  | **RI** | **NRM** | **LFS** | **OS** | **GRFS** | **acute GVHD II-IV** | **acute GVHD III-IV** | **chronic GVHD** | **extensive cGVHD** |
| --- | --- | --- | --- | --- | --- | --- | --- | --- | --- | --- |
| MRD at transplantation | MRD neg | 23.9% [21.8-26.1] | 18% [16.1-19.9] | 58.1% [55.6-60.7] | 67.3% [64.9-69.8] | 45% [42.4-47.6] | 33% [30.8-35.2] | 8.5% [7.2-9.9] | 41.4% [38.8-44] | 20% [17.9-22.2] |
|  | MRD pos | 32.1% [28.9-35.3] | 18.4% [15.8-21.1] | 49.6% [46.1-53] | 60.6% [57.2-64.1] | 35.4% [32-38.7] | 35.8% [32.7-38.9] | 10.6% [8.7-12.7] | 39% [35.5-42.4] | 19.7% [17-22.7] |
|  | P | <0.001 | 0.634 | <0.001 | 0.003 | <0.001 | 0.126 | 0.090 | 0.322 | 0.499 |
| Patient age | Age < median | 27.6% [25-30.2] | 12.9% [11.1-14.9] | 59.5% [56.6-62.3] | 69.3% [66.5-72] | 45% [42-47.9] | 34.2% [31.6-36.8] | 9.1% [7.6-10.7] | 40.4% [37.4-43.3] | 20.3% [18-22.8] |
|  | Age > median | 26.1% [23.6-28.7] | 23.3% [20.9-25.7] | 50.7% [47.7-53.6] | 60.6% [57.7-63.5] | 38% [35.1-40.9] | 33.7% [31.2-36.3] | 9.4% [7.9-11.1] | 40.7% [37.7-43.6] | 19.5% [17.1-21.9] |
|  | P | 0.215 | <0.001 | <0.001 | <0.001 | 0.001 | 0.750 | 0.787 | 0.877 | 0.365 |
| Diagnosis | Ph neg B | 26.5% [22.8-30.3] | 14.2% [11.5-17.3] | 59.3% [55.1-63.5] | 67% [63-71.1] | 44.5% [40.3-48.7] | 35.6% [31.8-39.3] | 10.4% [8.2-13] | 40.7% [36.5-45] | 19.2% [15.9-22.8] |
|  | Ph pos B | 26.7% [24.3-29.2] | 21.1% [18.9-23.4] | 52.1% [49.3-54.9] | 64.7% [62-67.4] | 39.4% [36.6-42.2] | 33.1% [30.7-35.6] | 9% [7.6-10.6] | 40.9% [38.1-43.8] | 20.3% [18-22.6] |
|  | T ALL | 27.5% [23.7-31.5] | 14.7% [11.8-17.9] | 57.8% [53.4-62.1] | 63.2% [58.9-67.5] | 43.4% [38.9-47.9] | 34.3% [30.4-38.2] | 8.5% [6.4-11.1] | 39.1% [34.6-43.5] | 19.7% [16.2-23.5] |
|  | P | 0.905 | <0.001 | <0.001 | 0.430 | 0.022 | 0.624 | 0.498 | 0.634 | 0.864 |
| Patient sex | Male | 28.6% [26.3-31] | 18.2% [16.2-20.2] | 53.2% [50.6-55.8] | 63.3% [60.8-65.9] | 39.7% [37.1-42.4] | 34.9% [32.6-37.2] | 10.2% [8.8-11.8] | 40.6% [37.9-43.2] | 19.6% [17.5-21.9] |
|  | Female | 24% [21.3-26.9] | 18% [15.6-20.6] | 57.9% [54.7-61.2] | 67.3% [64.2-70.4] | 44.2% [40.8-47.5] | 32.5% [29.7-35.4] | 7.7% [6.2-9.5] | 40.5% [37.1-43.8] | 20.3% [17.6-23.2] |
|  | P | 0.013 | 0.912 | 0.035 | 0.076 | 0.055 | 0.243 | 0.037 | 0.949 | 0.490 |
| KPS at transplantation | < 90% | 25.6% [21.5-29.8] | 18.3% [14.9-22.1] | 56.1% [51.4-60.8] | 63.5% [58.9-68.2] | 39.7% [34.9-44.5] | 36.7% [32.5-40.9] | 10.5% [8-13.4] | 43.5% [38.6-48.3] | 20.3% [16.4-24.4] |
|  | >= 90% | 25.9% [23.7-28.2] | 18% [16-20] | 56.1% [53.6-58.7] | 66% [63.5-68.5] | 43.4% [40.8-46] | 32.8% [30.5-35] | 8.8% [7.4-10.2] | 39.5% [36.8-42.1] | 18.8% [16.8-21] |
|  | P | 0.528 | 0.707 | 0.453 | 0.825 | 0.608 | 0.199 | 0.261 | 0.285 | 0.803 |
| Year of transplantation | Year < median | 29.5% [27-32] | 18.5% [16.5-20.7] | 52% [49.2-54.7] | 62.4% [59.7-65.1] | 38.4% [35.7-41.1] | 35.1% [32.6-37.7] | 10.1% [8.5-11.9] | 42.1% [39.3-44.9] | 20.9% [18.6-23.3] |
|  | Year > median | 23.2% [20.6-25.8] | 17.5% [15.3-19.9] | 59.3% [56.2-62.4] | 68.6% [65.6-71.5] | 45.5% [42.3-48.6] | 32.8% [30.3-35.3] | 8.4% [6.9-10] | 38.6% [35.5-41.6] | 18.7% [16.2-21.2] |
|  | P | 0.027 | 0.804 | 0.029 | 0.081 | 0.057 | 0.128 | 0.117 | 0.086 | 0.907 |
| Disease status at | CR1 | 24.9% [23-26.8] | 17.9% [16.2-19.5] | 57.3% [55.1-59.5] | 67.3% [65.2-69.4] | 43.2% [41-45.5] | 33.4% [31.5-35.4] | 8.7% [7.6-9.9] | 41.4% [39.1-43.6] | 20.4% [18.6-22.3] |
| transplantation | CR2 | 40.5% [34.8-46.2] | 19.8% [15.5-24.4] | 39.7% [33.9-45.4] | 47.9% [41.8-54] | 29.4% [23.9-34.9] | 37.5% [32.3-42.7] | 12.9% [9.6-16.9] | 34.7% [28.9-40.5] | 16.3% [12-21.1] |
|  | P | <0.001 | 0.298 | <0.001 | <0.001 | <0.001 | 0.170 | 0.015 | 0.027 | 0.106 |
| Donor | MSD | 30.5% [28-33.1] | 15.8% [13.9-17.9] | 53.6% [50.9-56.4] | 64.7% [62-67.4] | 38.9% [36.1-41.7] | 31.2% [28.9-33.6] | 8.4% [7-9.9] | 42% [39.1-44.8] | 21.8% [19.5-24.3] |
|  | UD 10/10 | 22.5% [19.6-25.6] | 20.1% [17.3-23] | 57.4% [53.9-61] | 66% [62.6-69.5] | 44% [40.4-47.7] | 36.5% [33.2-39.8] | 11.1% [9.1-13.4] | 39.3% [35.7-42.9] | 17.9% [15.2-20.9] |
|  | UD 9/10 | 21.3% [16.6-26.3] | 23.7% [18.9-28.8] | 55.1% [49.2-61] | 62.6% [56.8-68.4] | 46.6% [40.6-52.6] | 40% [34.5-45.4] | 8.4% [5.6-11.9] | 37.4% [31.6-43.3] | 16.6% [12.3-21.5] |
|  | P | <0.001 | 0.002 | 0.521 | 0.529 | 0.187 | 0.001 | 0.078 | 0.372 | 0.062 |
| Source of stem cells | BM | 26.6% [23-30.3] | 16.6% [13.8-19.7] | 56.8% [52.7-60.8] | 66.9% [63-70.8] | 45.6% [41.5-49.7] | 34.3% [30.6-38] | 8.9% [6.9-11.3] | 33.9% [29.9-37.9] | 15.7% [12.7-18.9] |
|  | Blood | 26.9% [24.8-29] | 18.6% [16.9-20.5] | 54.5% [52.1-56.9] | 64.3% [61.9-66.6] | 40.1% [37.7-42.5] | 33.9% [31.8-35.9] | 9.3% [8.1-10.7] | 42.8% [40.4-45.3] | 21.3% [19.3-23.4] |
|  | P | 0.646 | 0.046 | 0.261 | 0.232 | 0.004 | 0.642 | 0.791 | <0.001 | <0.001 |
| Donor sex | donor male | 29.7% [27.3-32.1] | 16.7% [14.8-18.6] | 53.7% [51.1-56.3] | 64.7% [62.1-67.2] | 40.3% [37.7-42.9] | 33.6% [31.3-35.9] | 9.8% [8.4-11.3] | 36.9% [34.3-39.5] | 18.4% [16.3-20.6] |
|  | donor female | 22.4% [19.6-25.2] | 20.4% [17.8-23.2] | 57.2% [53.9-60.6] | 65.4% [62.2-68.6] | 43.1% [39.7-46.5] | 34.7% [31.7-37.6] | 8.6% [6.9-10.4] | 47.2% [43.6-50.6] | 22.7% [19.8-25.7] |
|  | P | <0.001 | 0.029 | 0.09 | 0.515 | 0.579 | 0.644 | 0.312 | <0.001 | <0.001 |
| Female to male | no F->M | 27.8% [25.7-29.9] | 17.5% [15.8-19.2] | 54.8% [52.5-57.1] | 65.1% [62.8-67.3] | 41.7% [39.4-44.1] | 33.9% [31.8-35.9] | 9.2% [8-10.6] | 38.4% [36-40.7] | 18.9% [17.1-20.9] |
|  | F->M | 23.8% [20.2-27.7] | 20.4% [17-24.1] | 55.7% [51.3-60.2] | 64.2% [59.8-68.5] | 39.9% [35.4-44.3] | 34.7% [30.7-38.6] | 9.6% [7.3-12.2] | 49.3% [44.6-53.9] | 23.9% [20-28.1] |
|  | P | 0.113 | 0.179 | 0.656 | 0.782 | 0.368 | 0.805 | 0.804 | <0.001 | 0.002 |
| Patient CMV | CMV neg. | 26% [23.1-29.1] | 16.6% [14.2-19.2] | 57.3% [54-60.7] | 67.6% [64.4-70.9] | 43.2% [39.8-46.7] | 35.3% [32.3-38.4] | 10.2% [8.3-12.3] | 40.4% [37-43.8] | 19.6% [16.9-22.5] |
|  | CMV pos. | 27.3% [24.9-29.7] | 18.8% [16.8-20.9] | 53.9% [51.2-56.5] | 63.2% [60.5-65.8] | 40.2% [37.5-42.9] | 32.9% [30.6-35.2] | 8.9% [7.5-10.3] | 40.8% [38-43.5] | 19.9% [17.7-22.2] |
|  | P | 0.745 | 0.099 | 0.010 | 0.003 | 0.292 | 0.179 | 0.270 | 0.825 | 0.552 |
| Donor CMV | CMV neg. | 24.6% [22-27.3] | 18.9% [16.5-21.3] | 56.5% [53.4-59.6] | 66.7% [63.8-69.7] | 43.6% [40.5-46.8] | 35% [32.3-37.8] | 9.4% [7.8-11.2] | 38.3% [35.2-41.4] | 17.9% [15.5-20.4] |
|  | CMV pos. | 28.5% [25.9-31.1] | 17.3% [15.3-19.5] | 54.2% [51.3-57.1] | 63.7% [60.9-66.6] | 39.8% [37-42.7] | 33.5% [31-36] | 9.4% [7.9-11.1] | 42.4% [39.5-45.4] | 21.4% [19-23.9] |
|  | P | 0.023 | 0.456 | 0.198 | 0.215 | 0.034 | 0.361 | 0.984 | 0.055 | 0.008 |
| Conditioning based on | Chemotherapy | 35.2% [31-39.4] | 18.9% [15.7-22.3] | 45.9% [41.5-50.3] | 60% [55.6-64.4] | 37% [32.6-41.4] | 31% [27.3-34.7] | 8% [6-10.5] | 31.7% [27.6-36] | 14.6% [11.6-18.1] |
|  | TBI | 24.5% [22.5-26.5] | 17.9% [16.2-19.7] | 57.7% [55.3-60] | 66.3% [64.1-68.5] | 42.7% [40.4-45.1] | 34.8% [32.8-36.9] | 9.6% [8.3-11] | 43% [40.6-45.3] | 21.4% [19.4-23.4] |
|  | P | <0.001 | 0.222 | <0.001 | <0.001 | 0.017 | 0.034 | 0.215 | <0.001 | <0.001 |
| In vivo T-cell depletion | no *in vivo* TCD | 28.3% [26-30.7] | 17.7% [15.8-19.7] | 54% [51.3-56.6] | 64.5% [61.9-67.1] | 38.4% [35.8-41] | 34% [31.7-36.4] | 10.1% [8.7-11.7] | 44.5% [41.8-47.2] | 23% [20.7-25.3] |
|  | *in vivo* TCD | 24.8% [21.9-27.7] | 18.5% [16-21.2] | 56.7% [53.4-60] | 65.6% [62.4-68.9] | 46.2% [42.8-49.6] | 33.9% [30.9-36.8] | 8.3% [6.6-10.1] | 33.7% [30.4-36.9] | 14.8% [12.4-17.3] |
|  | P | 0.362 | 0.930 | 0.461 | 0.570 | <0.001 | 0.915 | 0.112 | <0.001 | <0.001 |
