# Supplementary material for: Measurable residual disease at myeloablative allogeneic transplantation in adults with acute lymphoblastic leukemia: a retrospective registry study on 2780 patients from the acute leukemia working party of the EBMT
Source: J Hematol Oncol. 2019 Oct 23;12:108. doi: 10.1186/s13045-019-0790-x (PMC6813121; doi:10.1186/s13045-019-0790-x)
Supplement: Supplementary file 3 — Additional file 3. A. Univariate planned sub-analyses performed separately in subgroups of patients transplanted after chemotherapy-based and TBI-based conditioning. Abbreviations: GVHD, graft-versus-host disease; GRFS, GVHD-free and relapse-free survival; LFS, leukemia free survival; MRD, measurable residual disease; NRM, non-relapse mortality; OS, overall survival; RI, relapse incidence. B. Multivariate planned sub-analyses performed separately in subgroups of patients transplanted after chemotherapy-based conditioning (571 patients of whom 382 were MRD negative and 205 MRD positive) and TBI-based conditioning (1943 patients of whom 1278 were MRD negative and 680 MRD positive). Abbreviations: BM, bone marrow; CR, complete remission; CMV, cytomegalovirus; GVHD, graft-versus-host disease; GRFS, GVHD-free and relapse-free survival; KPS, Karnofsky performance score; LFS, leukemia free survival; MRD, measurable residual disease; NRM, non-relapse mortality; OS, overall survival; Ph, Philadelphia chromosome/BCR-ABL gene rearrangement; RI, relapse incidence; TCD, T-cell depletion; UD, unrelated donor. [file 13045_2019_790_MOESM3_ESM.docx]

Additional file **3:**

A. Univariate planned sub-analyses performed separately in subgroups of patients transplanted after chemotherapy-based and TBI-based conditioning. Abbreviations: GVHD, graft-versus-host disease; GRFS, GVHD-free and relapse-free survival; LFS, leukemia free survival; MRD, measurable residual disease; NRM, non-relapse mortality; OS, overall survival; RI, relapse incidence.

|  |  | **RI** | **NRM** | **LFS** | **OS** | **GRFS** | **acute GVHD II-IV** | **acute GVHD III-IV** | **chronic GVHD** | **extensive cGVHD** |
| --- | --- | --- | --- | --- | --- | --- | --- | --- | --- | --- |
| **Chemotherapy-based** | MRD negative | 33.7% [28.6-38.9] | 18.3% [14.4-22.5] | 48% [42.5-53.5] | 62.2% [56.8-67.7] | 38% [32.4-43.6] | 29% [24.6-33.6] | 7.1% [4.7-10] | 33.1% [27.9-38.4] | 15.2% [11.3-19.5] |
| **conditioning** | MRD positive | 37.8% [30.7-44.9] | 20% [14.6-26] | 42.2% [34.9-49.5] | 56% [48.5-63.4] | 35.2% [28-42.4] | 34.8% [28.4-41.2] | 9.8% [6.2-14.4] | 29.1% [22.3-36.2] | 13.6% [8.8-19.5] |
|  | P | 0.089 | 0.486 | 0.026 | 0.311 | 0.078 | 0.171 | 0.267 | 0.219 | 0.443 |
| **TBI-based conditioning** | MRD negative | 21.2% [18.8-23.6] | 17.8% [15.7-20.1] | 61% [58.2-63.9] | 68.7% [66-71.5] | 46.8% [43.9-49.8] | 34.2% [31.6-36.7] | 8.9% [7.5-10.6] | 43.7% [40.7-46.7] | 21.4% [18.9-23.9] |
|  | MRD positive | 30.4% [26.9-34] | 17.9% [15.1-20.9] | 51.6% [47.7-55.6] | 62% [58.1-65.8] | 35.4% [31.6-39.2] | 36.1% [32.6-39.6] | 10.8% [8.6-13.3] | 41.7% [37.7-45.6] | 21.4% [18.2-24.8] |
|  | P | <0.001 | 0.855 | <0.001 | 0.004 | <0.001 | 0.306 | 0.178 | 0.599 | 0.654 |
|  |  | **RI** | **NRM** | **LFS** | **OS** | **GRFS** | **acute GVHD II-IV** | **acute GVHD III-IV** | **chronic GVHD** | **extensive cGVHD** |
| **MRD negative** | Chemotherapy-based | 33.7% [28.6-38.9] | 18.3% [14.4-22.5] | 48% [42.5-53.5] | 62.2% [56.8-67.7] | 38% [32.4-43.6] | 29% [24.6-33.6] | 7.1% [4.7-10] | 33.1% [27.9-38.4] | 15.2% [11.3-19.5] |
|  | TBI-based conditioning | 21.2% [18.8-23.6] | 17.8% [15.7-20.1] | 61% [58.2-63.9] | 68.7% [66-71.5] | 46.8% [43.9-49.8] | 34.2% [31.6-36.7] | 8.9% [7.5-10.6] | 43.7% [40.7-46.7] | 21.4% [18.9-23.9] |
|  | P | <0.001 | 0.497 | <0.001 | <0.001 | 0.029 | 0.034 | 0.233 | 0.008 | 0.006 |
| **MRD positive** | Chemotherapy-based | 37.8% [30.7-44.9] | 20% [14.6-26] | 42.2% [34.9-49.5] | 56% [48.5-63.4] | 35.2% [28-42.4] | 34.8% [28.4-41.2] | 9.8% [6.2-14.4] | 29.1% [22.3-36.2] | 13.6% [8.8-19.5] |
|  | TBI-based conditioning | 30.4% [26.9-34] | 17.9% [15.1-20.9] | 51.6% [47.7-55.6] | 62% [58.1-65.8] | 35.4% [31.6-39.2] | 36.1% [32.6-39.6] | 10.8% [8.6-13.3] | 41.7% [37.7-45.6] | 21.4% [18.2-24.8] |
|  | P | 0.006 | 0.259 | <0.001 | 0.015 | 0.295 | 0.469 | 0.605 | 0.003 | 0.010 |

B. Multivariate planned sub-analyses performed separately in subgroups of patients transplanted after chemotherapy-based conditioning (571 patients of whom 382 were MRD negative and 205 MRD positive) and TBI-based conditioning (1943 patients of whom 1278 were MRD negative and 680 MRD positive). Abbreviations: BM, bone marrow; CR, complete remission; CMV, cytomegalovirus; GVHD, graft-versus-host disease; GRFS, GVHD-free and relapse-free survival; KPS, Karnofsky performance score; LFS, leukemia free survival; MRD, measurable residual disease; NRM, non-relapse mortality; OS, overall survival; Ph, Philadelphia chromosome/BCR-ABL gene rearrangement; RI, relapse incidence; TCD, T-cell depletion; UD, unrelated donor.

| **Chemotherapy-based conditioning** | **RI** | | **NRM** | | **LFS** | | **OS** | | **GRFS** | | **acute GVHD II-IV** | | **acute GVHD III-IV** | | **chronic GVHD** | | **extensive cGVHD** | |
| --- | --- | --- | --- | --- | --- | --- | --- | --- | --- | --- | --- | --- | --- | --- | --- | --- | --- | --- |
|  | HR (95% CI) | P | HR (95% CI) | P | HR (95% CI) | P | HR (95% CI) | P | HR (95% CI) | P | HR (95% CI) | P | HR (95% CI) | P | HR (95% CI) | P | HR (95% CI) | P |
| MRD positive vs neg | 1.58 (1.13-2.21) | 0.008 | 0.87 (0.56-1.35) | 0.534 | 1.25 (0.96-1.63) | 0.093 | 1.04 (0.77-1.40) | 0.806 | 1.21 (0.94-1.56) | 0.143 | 1.27 (0.89-1.82) | 0.184 | 1.06 (0.51-2.22) | 0.870 | 0.67 (0.43-1.04) | 0.075 | 0.70 (0.37-1.30) | 0.254 |
| Ph neg B-ALL (reference) | 1 | - | 1 | - | 1 | - | 1 | - | 1 | - | 1 | - | 1 | - | 1 | - | 1 | - |
| Ph positive B-ALL | 0.98 (0.64-1.52) | 0.940 | 1.21 (0.69-2.14) | 0.504 | 1.08 (0.76-1.52) | 0.669 | 0.99 (0.68-1.45) | 0.964 | 0.99 (0.72-1.37) | 0.972 | 0.77 (0.50-1.20) | 0.244 | 0.64 (0.26-1.57) | 0.330 | 0.63 (0.38-1.05) | 0.075 | 0.77 (0.39-1.55) | 0.466 |
| T-ALL | 1.32 (0.78-2.23) | 0.306 | 0.80 (0.37-1.74) | 0.572 | 1.10 (0.71-1.71) | 0.656 | 1.15 (0.72-1.83) | 0.557 | 1.00 (0.66-1.52) | 0.988 | 1.27 (0.75-2.16) | 0.381 | 1.54 (0.55-4.28) | 0.412 | 0.57 (0.29-1.13) | 0.107 | 0.59 (0.21-1.63) | 0.305 |
| Age (per 10 years) | 0.99 (0.87-1.14) | 0.983 | 1.27 (1.08-1.50) | 0.004 | 1.09 (0.99-1.21) | 0.089 | 1.10 (0.98-1.24) | 0.091 | 1.07 (0.97-1.18) | 0.207 | 1.04 (0.91-1.20) | 0.533 | 1.13 (0.84-1.53) | 0.428 | 1.15 (0.98-1.36) | 0.094 | 1.03 (0.82-1.30) | 0.773 |
| Year of transplant | 0.96 (0.91-1.01) | 0.082 | 0.95 (0.89-1.01) | 0.089 | 0.95 (0.92-0.99) | 0.018 | 0.96 (0.92-1.01) | 0.087 | 0.97 (0.93-1.00) | 0.063 | 0.95 (0.90-1.00) | 0.045 | 0.96 (0.86-1.07) | 0.435 | 0.93 (0.87-0.99) | 0.030 | 0.99 (0.89-1.08) | 0.753 |
| CR2 vs CR1 | 1.88 (1.22-2.89) | 0.004 | 1.94 (1.12-3.36) | 0.018 | 1.88 (1.34-2.64) | <0.001 | 2.07 (1.44-2.98) | <0.001 | 2.04 (1.48-2.81) | <0.001 | 1.10 (0.70-1.74) | 0.671 | 2.70 (1.17-6.20) | 0.020 | 1.27 (0.70-2.31) | 0.425 | 2.85 (1.33-6.09) | 0.007 |
| KPS >=90% | 1.09 (0.72-1.65) | 0.672 | 0.94 (0.57-1.55) | 0.816 | 1.02 (0.74-1.40) | 0.917 | 0.86 (0.61-1.21) | 0.396 | 0.93 (0.69-1.25) | 0.617 | 0.98 (0.64-1.48) | 0.909 | 1.59 (0.65-3.91) | 0.314 | 1.02 (0.61-1.69) | 0.947 | 0.64 (0.33-1.27) | 0.202 |
| UD 10/10 | 0.79 (0.52-1.20) | 0.261 | 2.02 (1.20-3.40) | 0.008 | 1.15 (0.83-1.59) | 0.414 | 1.23 (0.87-1.75) | 0.244 | 1.10 (0.81-1.51) | 0.535 | 1.11 (0.71-1.73) | 0.644 | 1.10 (0.43-2.82) | 0.835 | 1.31 (0.79-2.19) | 0.300 | 0.99 (0.48-2.04) | 0.970 |
| UD 9/10 | 0.48 (0.25-0.94) | 0.031 | 2.16 (1.04-4.46) | 0.038 | 0.89 (0.55-1.44) | 0.635 | 1.10 (0.65-1.87) | 0.726 | 0.94 (0.60-1.47) | 0.770 | 1.48 (0.83-2.64) | 0.183 | 0.95 (0.26-3.44) | 0.937 | 1.30 (0.63-2.68) | 0.477 | 1.22 (0.43-3.43) | 0.710 |
| Blood vs BM | 1.14 (0.75-1.72) | 0.535 | 1.46 (0.83-2.58) | 0.186 | 1.25 (0.90-1.75) | 0.185 | 1.54 (1.04-2.28) | 0.033 | 1.42 (1.03-1.96) | 0.032 | 1.38 (0.85-2.26) | 0.197 | 1.33 (0.46-3.80) | 0.598 | 1.16 (0.69-1.94) | 0.575 | 2.00 (0.89-4.50) | 0.093 |
| Female vs male | 0.78 (0.55-1.10) | 0.152 | 0.83 (0.54-1.27) | 0.384 | 0.79 (0.61-1.03) | 0.087 | 0.74 (0.55-1.00) | 0.048 | 0.78 (0.61-1.01) | 0.056 | 0.95 (0.66-1.35) | 0.754 | 0.57 (0.26-1.23) | 0.149 | 0.72 (0.47-1.09) | 0.118 | 0.81 (0.44-1.46) | 0.475 |
| Donor female vs male | 0.64 (0.45-0.91) | 0.014 | 1.06 (0.69-1.62) | 0.800 | 0.77 (0.59-1.01) | 0.062 | 0.90 (0.67-1.21) | 0.469 | 0.75 (0.58-0.98) | 0.032 | 1.14 (0.80-1.61) | 0.474 | 0.62 (0.28-1.35) | 0.227 | 1.32 (0.88-1.98) | 0.184 | 1.18 (0.66-2.12) | 0.580 |
| Patient CMV pos vs neg | 0.99 (0.67-1.45) | 0.943 | 0.94 (0.58-1.53) | 0.797 | 0.96 (0.71-1.30) | 0.790 | 1.17 (0.83-1.64) | 0.371 | 0.96 (0.72-1.28) | 0.774 | 1.00 (0.67-1.50) | 0.988 | 1.93 (0.78-4.77) | 0.154 | 0.75 (0.48-1.19) | 0.218 | 0.65 (0.34-1.24) | 0.192 |
| Donor CMV pos vs neg | 1.01 (0.69-1.46) | 0.977 | 1.52 (0.95-2.43) | 0.080 | 1.18 (0.89-1.58) | 0.254 | 1.21 (0.87-1.66) | 0.255 | 1.07 (0.81-1.41) | 0.638 | 1.06 (0.72-1.56) | 0.757 | 0.98 (0.44-2.22) | 0.967 | 1.31 (0.83-2.06) | 0.253 | 1.20 (0.63-2.29) | 0.570 |
| In vivo TCD vs no TCD | 0.90 (0.60-1.34) | 0.592 | 0.67 (0.41-1.11) | 0.119 | 0.79 (0.58-1.09) | 0.149 | 0.79 (0.56-1.11) | 0.173 | 0.84 (0.63-1.14) | 0.261 | 1.04 (0.69-1.58) | 0.837 | 0.86 (0.36-2.09) | 0.742 | 0.73 (0.44-1.21) | 0.226 | 0.73 (0.36-1.49) | 0.385 |
| Center (frailty) | - | 0.914 | - | 0.283 | - | 0.908 | - | 0.910 | - | 0.901 | - | 0.224 | - | 0.031 | - | 0.035 | - | 0.205 |

| **TBI-based**  **conditioning** | **RI** | | **NRM** | | **LFS** | | **OS** | | **GRFS** | | **acute GVHD II-IV** | | **acute GVHD III-IV** | | **chronic GVHD** | | **extensive cGVHD** | |
| --- | --- | --- | --- | --- | --- | --- | --- | --- | --- | --- | --- | --- | --- | --- | --- | --- | --- | --- |
|  | HR (95% CI) | P | HR (95% CI) | P | HR (95% CI) | P | HR (95% CI) | P | HR (95% CI) | P | HR (95% CI) | P | HR (95% CI) | P | HR (95% CI) | P | HR (95% CI) | P |
| MRD positive vs neg | 1.53 (1.23-1.90) | <0.001 | 1.04 (0.81-1.35) | 0.746 | 1.30 (1.10-1.53) | 0.002 | 1.26 (1.05-1.51) | 0.012 | 1.28 (1.11-1.47) | <0.001 | 1.12 (0.93-1.35) | 0.222 | 1.11 (0.79-1.56) | 0.560 | 1.07 (0.90-1.28) | 0.443 | 1.08 (0.85-1.38) | 0.540 |
| Ph neg B-ALL (reference) | 1 | - | 1 | - | 1 | - | 1 | - | 1 | - | 1 | - | 1 | - | 1 | - | 1 | - |
| Ph positive B-ALL | 0.90 (0.69-1.19) | 0.459 | 1.54 (1.10-2.14) | 0.012 | 1.12 (0.91-1.38) | 0.289 | 0.95 (0.76-1.19) | 0.659 | 1.01 (0.85-1.21) | 0.901 | 1.02 (0.82-1.28) | 0.838 | 0.92 (0.62-1.38) | 0.694 | 1.04 (0.83-1.29) | 0.752 | 1.12 (0.83-1.50) | 0.470 |
| T-ALL | 1.04 (0.76-1.41) | 0.806 | 1.25 (0.84-1.86) | 0.276 | 1.12 (0.88-1.42) | 0.369 | 1.07 (0.82-1.38) | 0.621 | 1.02 (0.83-1.25) | 0.876 | 1.07 (0.82-1.39) | 0.614 | 0.72 (0.44-1.19) | 0.201 | 0.90 (0.69-1.16) | 0.399 | 1.08 (0.76-1.54) | 0.656 |
| Age (per 10 years) | 1.05 (0.96-1.15) | 0.267 | 1.32 (1.21-1.44) | <0.001 | 1.17 (1.10-1.24) | <0.001 | 1.23 (1.15-1.31) | <0.001 | 1.13 (1.07-1.20) | <0.001 | 1.07 (1.00-1.15) | 0.054 | 1.08 (0.94-1.23) | 0.269 | 1.06 (0.99-1.13) | 0.099 | 1.06 (0.97-1.17) | 0.191 |
| Year of transplant | 0.98 (0.95-1.01) | 0.185 | 0.98 (0.95-1.01) | 0.244 | 0.98 (0.96-1.00) | 0.108 | 0.98 (0.96-1.01) | 0.173 | 0.99 (0.97-1.01) | 0.339 | 0.98 (0.96-1.01) | 0.177 | 0.97 (0.93-1.01) | 0.169 | 0.97 (0.94-0.99) | 0.007 | 1.01 (0.97-1.04) | 0.729 |
| CR2 vs CR1 | 2.54 (1.92-3.37) | <0.001 | 1.53 (1.05-2.23) | 0.029 | 2.09 (1.67-2.61) | <0.001 | 2.11 (1.65-2.70) | <0.001 | 1.56 (1.27-1.92) | <0.001 | 1.23 (0.95-1.60) | 0.119 | 1.38 (0.86-2.22) | 0.187 | 1.00 (0.74-1.35) | 0.998 | 0.89 (0.57-1.39) | 0.606 |
| KPS >=90% | 1.10 (0.86-1.42) | 0.448 | 1.20 (0.90-1.61) | 0.209 | 1.15 (0.95-1.39) | 0.151 | 1.08 (0.88-1.33) | 0.477 | 1.07 (0.91-1.26) | 0.409 | 0.97 (0.79-1.19) | 0.762 | 0.89 (0.62-1.28) | 0.532 | 1.02 (0.84-1.24) | 0.827 | 1.18 (0.90-1.55) | 0.228 |
| UD 10/10 | 0.59 (0.44-0.80) | <0.001 | 1.95 (1.41-2.68) | <0.001 | 1.01 (0.81-1.25) | 0.960 | 1.25 (0.98-1.58) | 0.072 | 1.17 (0.97-1.42) | 0.096 | 1.86 (1.46-2.35) | <0.001 | 2.40 (1.58-3.64) | <0.001 | 1.43 (1.15-1.78) | 0.001 | 1.39 (1.03-1.87) | 0.031 |
| UD 9/10 | 0.59 (0.41-0.87) | 0.007 | 2.23 (1.49-3.33) | <0.001 | 1.08 (0.82-1.43) | 0.564 | 1.44 (1.07-1.94) | 0.017 | 1.06 (0.83-1.36) | 0.631 | 1.81 (1.34-2.45) | <0.001 | 2.04 (1.15-3.62) | 0.015 | 1.34 (1.00-1.80) | 0.052 | 1.14 (0.75-1.75) | 0.536 |
| PB vs BM | 0.81 (0.63-1.04) | 0.105 | 0.96 (0.72-1.29) | 0.798 | 0.86 (0.71-1.04) | 0.115 | 0.79 (0.64-0.97) | 0.027 | 1.13 (0.95-1.34) | 0.173 | 1.02 (0.82-1.28) | 0.836 | 1.07 (0.73-1.59) | 0.725 | 1.50 (1.21-1.87) | <0.001 | 1.81 (1.33-2.47) | <0.001 |
| Female vs male | 0.83 (0.67-1.04) | 0.108 | 1.01 (0.79-1.30) | 0.935 | 0.90 (0.77-1.07) | 0.239 | 0.95 (0.79-1.14) | 0.547 | 0.90 (0.78-1.04) | 0.155 | 0.95 (0.80-1.14) | 0.605 | 0.78 (0.55-1.10) | 0.159 | 0.97 (0.81-1.15) | 0.704 | 0.93 (0.73-1.17) | 0.530 |
| Donor female vs male | 0.66 (0.52-0.83) | <0.001 | 1.37 (1.07-1.76) | 0.012 | 0.93 (0.79-1.10) | 0.374 | 0.98 (0.82-1.18) | 0.819 | 1.03 (0.89-1.19) | 0.711 | 1.11 (0.92-1.33) | 0.282 | 1.13 (0.80-1.60) | 0.500 | 1.36 (1.15-1.62) | <0.001 | 1.25 (0.99-1.58) | 0.058 |
| Patient CMV pos vs neg | 0.89 (0.70-1.13) | 0.331 | 1.39 (1.06-1.82) | 0.018 | 1.10 (0.92-1.32) | 0.284 | 1.31 (1.07-1.60) | 0.008 | 1.04 (0.89-1.21) | 0.658 | 0.95 (0.79-1.15) | 0.614 | 0.91 (0.64-1.30) | 0.595 | 1.05 (0.87-1.27) | 0.618 | 1.09 (0.84-1.42) | 0.502 |
| Donor CMV pos vs neg | 1.18 (0.93-1.50) | 0.179 | 0.73 (0.56-0.95) | 0.021 | 0.93 (0.78-1.11) | 0.448 | 0.86 (0.71-1.05) | 0.138 | 1.09 (0.94-1.27) | 0.264 | 1.09 (0.90-1.32) | 0.363 | 1.15 (0.80-1.65) | 0.446 | 1.16 (0.96-1.4) | 0.119 | 1.34 (1.03-1.74) | 0.028 |
| In vivo TCD vs no TCD | 1.38 (1.03-1.85) | 0.031 | 0.66 (0.48-0.90) | 0.008 | 0.97 (0.79-1.21) | 0.801 | 0.84 (0.66-1.06) | 0.139 | 0.72 (0.59-0.87) | <0.001 | 0.66 (0.52-0.83) | <0.001 | 0.43 (0.28-0.65) | <0.001 | 0.55 (0.44-0.69) | <0.001 | 0.43 (0.32-0.59) | <0.001 |
| Center (frailty) |  | 0.069 |  | 0.127 | - | 0.054 | - | 0.008 | - | 0.034 | - | <0.001 | - | 0.037 | - | 0.080 | - | 0.013 |
